# Elevated CEA is associated with worse survival in recurrent rectal cancer

**DOI:** 10.18632/oncotarget.22511

**Published:** 2017-11-18

**Authors:** Won Kyung Cho, Doo Ho Choi, Hee Chul Park, Won Park, Jeong Il Yu, Young Suk Park, Joon Oh Park, Ho Yeong Lim, Won Ki Kang, Hee Cheol Kim, Yong Beom Cho, Seong Hyeon Yun, Woo Yong Lee

**Affiliations:** ^1^ Department of Radiation Oncology, Seoul, Republic of Korea; ^2^ Division of Hematology-Oncology, Department of Medicine, Seoul, Republic of Korea; ^3^ Department of Surgery, Samsung Medical Center, Sungkyunkwan University School of Medicine, Seoul, Republic of Korea

**Keywords:** CEA, rectal cancer, recurrence, survival

## Abstract

This study investigated the prognostic impact of serum carcinoembryonic antigen (CEA) level in recurrent rectal cancer. We reviewed 745 patients who developed recurrence after curative treatment for rectal cancer between January 2000 and December 2012. Multivariate analyses for survival revealed that age > 60 years (*p* = 0.005), r-CEA ≥ 5 ng/ml (*p* < 0.001), disease free interval (DFI) < 12 months (*p* < 0.001), and palliative or conservative treatment (*p* < 0.001) were unfavorable factors.

## INTRODUCTION

Serum carcinoembryonic antigen (CEA) is one of the most widely used tumor markers for screening test, predicting treatment response and survival, and detecting recurrence in colorectal cancer [1–4]. CEA was first identified in human colon carcinoma tissue extracts and is a glycoprotein on the surface of colonic epithelial cells and known to play a critical role as a ligand in cancer dissemination [4]. Elevated serum CEA is found in 17∼47% of colorectal cancer patients [5, 6].

The prognostic impact of pretreatment CEA level (pre-CEA) of rectal cancer has been studied in several studies and pre-CEA of 5 ng/ml or higher is proven to be associated with poor prognosis [7–10]. Recently, a Korean multi-institutional study found that patients with elevated pre-CEA have lower recurrence-free survival (RFS) and overall survival (OS) than patients with normal pre-CEA (5-year RFS 74.2% vs. 63.5%, *p* < 0.001 and 5-year OS 86.9% vs. 81.8%, *p* = 0.001) among rectal cancer patients treated with neoadjuvant chemoradiotherapy (NACRT) and surgery, even after propensity score matching to control covariates including stage [11].

Clinical significance of not only pre-CEA but also post-NACRT CEA level, postoperative CEA level, and CEA ratio were investigated to predict prognosis of recurrent rectal cancer patients. Perez et al. [12] reported that post-NACRT CEA higher than 5 ng/ml is associated with lower rate of pathological response and worse disease-free survival (DFS). Jang et al. [13] reported that post-NACRT CEA 2.7 ng/mL or less is an independent predictor of good response. Kim et al. [14] noted that a reduction in the pre-CEA to post-NACRT CEA level is a favorable factor for survival in patients whose pre-CEA level was higher than 6 ng/ml.

CEA is also demonstrated as useful markers for detecting recurrence [1–4]. Most current guidelines recommend checking serum CEA regularly following treatment as a way to detect recurrent disease [1, 15]. However, prognostic impact of CEA at the time of recurrence (r-CEA) in recurrent rectal cancer has not been addressed. This study investigated the prognostic impact of elevated r-CEA on survival in recurrent rectal cancer.

## RESULTS

### Patient characteristics

The median age of all patients was 57 (range 19∼86) years and median disease-free interval (DFI) was 16.2 (0.7∼158.0) months. Of all patients, 360 (48.3%) patients were treated with surgery alone, 219 (29.4%) with NACRT followed by surgery, 158 (21.2%) with surgery followed by postoperative chemoradiotherapy, and 8 (1.0%) with preoperative radiotherapy and surgery. Among the all recurrent patients,479 (64.3%) patients had normal r-CEA and 266 (35.7%) presented r-CEA ≥ 5. The characteristics of patients according to CEA group are in Table 1. There was no difference in distribution of gender, initial T-stage, initial treatment modality, and patterns of failure between the groups. Initial pN2-3 was more frequent in groups in patients with elevated r-CEA level than those with normal r-CEA (41.7% vs. 28.2%, *p* < 0.001). Following recurrence, 165 (34.4%) of patients with normal r-CEA received salvage treatment while 54 (20.3%) of patients with elevated r-CEA received salvage treatment (*p* < 0.001).

**Table 1:** Patient characteristics

	r-CEA < 5 (n=479)	r-CEA ≥ 5 (n=266)	*p*
Median disease free interval (range, months)	16.4 (0.9-158.0)	15.8 (0.7-118.4)	
Gender			0.390
Male	314 (65.6%)	166 (62.4%)	
Female	165 (34.4%)	100 (37.6%)	
Median age (range, years)			
Initial pT-stage			0.089
pT0-2	116 (24.2%)	50 (18.8%)	
pT3-4	363 (75.8%)	216 (81.2%)	
Initial pN-stage			< 0.001
pN0-1	344 (71.8%)	155 (58.3%)	
pN2-3	135 (28.2%)	111 (41.7%)	
Initial CEA			< 0.001
CEA < 5	383 (80.0%)	118 (44.4%)	
CEA ≥ 5	96 (20.0%)	148 (55.6%)	
Initial treatment			0.899
Surgery alone	228 (47.6%)	132 (49.6%)	
Surgery + adjuvant CCRT	101 (21.1%)	57 (21.4%)	
NACRT + Surgery	144 (30.1%)	75 (28.2%)	
Preop RT + Surgery	6 (1.2%)	2 (0.8%)	
Pattern of failure			0.064
LRR	91 (19.0%)	51 (19.2%)	
DM	323 (67.4%)	162 (60.9%)	
LRR + DM	65 (13.6%)	53 (19.9%)	
Treatment after recurrence			< 0.001
Salvage treatment	165 (34.4%)	54 (20.3%)	
Surgery +/− CCRT	140 (29.2%)	37 (13.9%)	
RT +/− chemo	9 (1.9%)	10 (3.8%)	
RFA only (for liver metastasis)	16 (3.3%)	7 (2.6%)	
Palliative or conservative	248 (51.8%)	183 (68.8%)	
Unknown	66 (13.8%)	29 (10.9%)	

CEA, carcinoembryonic antigen level; CCRT, concurrent chemoradiotherapy; NACRT, neoadjuvant chemoradiotherapy; LRR, locoregional recurrence; DM, distant metastasis; RT, radiation therapy; RFA, radiofrequency ablation

### Prognostic factors for survival

**Figure 1:**
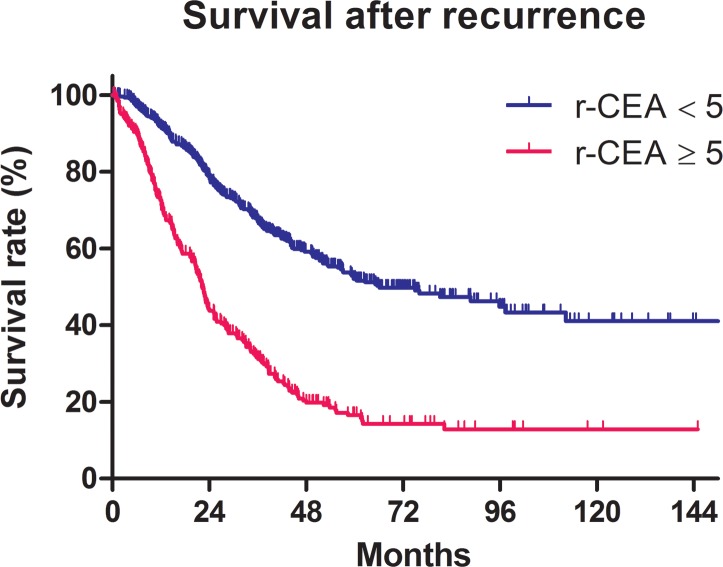
Survival after recurrence according to the CEA at the time of recurrence (r-CEA) level

Among the 745 patients, 404 (54.2%) died. At 5 years, OS from first recurrence was 39.8% for all patients and was significantly different between the patients with normal r-CEA and those with elevated r-CEA (52.1% vs. 16.6%, *p* < 0.001, Figure 1). The prognostic significance for survival was analyzed for gender, age, initial CEA, r-CEA, DFI, treatment after recurrence, and patterns of failure. Univariate analysis for OS showed that age > 60 (*p* < 0.001), initial CEA ≥ 5 ng/ml (*p* < 0.001), r-CEA ≥5 ng/ml (*p* < 0.001), DFI less than 12 months (*p* < 0.001), and palliative or conservative treatment (*p* < 0.001) are unfavorable prognostic factors (Table 2). The factors that had p-value less than 0.05 were included in multivariate analyses, which included age, initial CEA, r-CEA, and DFI. In multivariate analyses, significantly unfavorable factors for survival were age > 60 (hazard ratio (HR) = 1.419, 95% confidence interval (CI) = 1.164-1.731, *p* < 0.001), r-CEA ≥ 5 ng/ml (HR = 2.508, 95% CI = 2.014-3.124, *p* < 0.001), DFI < 12 months (HR = 1.634, 95% CI = 1.338-1.996, *p* < 0.001), and palliative or conservative treatment (HR = 2.709 95% CI = 2.119-3.463, *p* < 0.001, Table 2).

**Table 2:** Prognostic factors by uni- and multi-variate analysis for survival after recurrence

Characteristics	5 year survival	*Uni-*	*Multi-*	HR (95% CI)
Gender				
Male Female	39.9% 38.1%	*0.850*	*-*	-
Age				
≤ 60 years > 60 years	43.0% 33.2%	***< 0.001***	***0.001***	**1.419 (1.164-1.731)**
Initial CEA				
< 5 ng/ml ≥ 5 ng/ml	47.5% 22.9%	***< 0.001***	*0.439*	1.092 (0.874-1.364)
r-CEA				
< 5 ng/ml ≥ 5 ng/ml	52.1% 16.6%	***< 0.001***	***< 0.001***	**2.508 (2.014-3.124)**
Disease free interval				
< 12 months ≥12 months	26.9% 45.9%	***< 0.001***	***< 0.001***	**1.634 (1.338-1.996)**
Treatment after recurrence				
Salvage Tx Palliative/Conservative Tx	58.6% 24.1%	***< 0.001***	***< 0.001***	**2.709 (2.119-3.463)**
Pattern of failure				
Isolated LRR Distant metastasis	48.6% 37.3%	*0.094*	*-*	-

r-CEA, CEA at the time of recurrence; LRR, loco-regional recurrence; HR, hazard ratio; CI, confidence interval, Tx, treatment

## DISCUSSION

Serial assay for CEA after rectal cancer treatment is usually recommended and its sensitivity to detect recurrence is reported as 70–80% [15, 16]. However, evidence is limited regarding prognostic impact of elevated CEA in recurrent rectal cancer. Some patients with elevated CEA at diagnosis recur without r-CEA elevation and vice versa. Is the r-CEA also associated with remaining survival of the patients as initial CEA is associated with DFS in primary cancer? The current study found that CEA at the time of recurrence is related to survival after recurrence in recurrent rectal cancer. The 5-year survival rate following recurrence was significantly lower in patients with elevated r-CEA than the patients without elevation of r-CEA even in multivariate analysis including initial CEA (HR 2.508, 95% CI 2.014-3.124, *p* = 0.001, Table 2).

Several studies reported discrepancy between initial and recurrent CEA level in rectal cancer. Grossmann et al. indicated that 73 of 282 (25.9%) patients with recurrent disease who had initially normal CEA expressed high r-CEA level [15]. Another Korean study [17] found that 21.4% of patients who had normal CEA at diagnosis showed increased CEA when they recur. In this study, 23.5% (118/501) of patients who had initially normal CEA had elevated CEA at recurrence and 39.3% (96/244) of patients who had initially elevated CEA showed normal CEA at recurrence.

The reason why serum CEA is differentially expressed in initial and recurrent rectal cancer is unclear. It might be simply due to different tumor burden as shown in metastatic colon cancer study reporting relationship between CEA level and radiologic tumor burden [18]. On the other hand, initial and recurrent tumor might have different biology. Chang et al. failed to demonstrate that location of tumor involvement induces discrepancy between CEA level of primary and recurrent cancer [19]. Several literatures proposed possible explanations why high CEA level is associated with poor prognosis [20, 21]. Jessup et al. [20] suggested that CEA-producing tumors have higher tumorigenic potential and ability to spread distantly that might be facilitated by its role in cell adhesion. Recent experimental data suggest that an adoptive immune reaction of CEA-specific T-cells causes enteropathy, resulting in loss of mucosal integrity with increased epithelial leakage facilitating tumor growth or recurrence [21]. Whatever caused elevation of CEA (e.g., residual tumor burden or aggressive tumor biology), clinicians can expect poor survival of patients with elevated r-CEA level. If we can select the patients with short expected life accurately, we can spare aggressive treatment and focus on palliation since salvage treatment involves possibility of adverse effect as well as requires excessive medical costs. Although r-CEA alone is not enough for accurate calculation of expected survival, this study suggests that r-CEA is one of the key factors predicting survival in recurrent rectal cancer.

This study has a few limitations. First, we reviewed retrospective data after excluding patients for lack of information. To assess selection bias, we calculated overall survival of the patients with missing CEA levels at recurrence and compared with survival of the patients in the current study (2-yr OS 26.5% vs. 39.8%, *p* = 0.003). It is assumed that most of the patients with missing CEA level are lost to follow-up and lack of salvage treatment. Second, because of the long study period, systemic errors that we could not take into account might have occurred in measuring CEA according to time period. Furthermore, we did not consider heavy smoking or other factors such as liver disease that may elevate serum CEA level. However, whatever induced it, we demonstrated that elevated r-CEA is associated with poor survival in recurrent patients. Possibly, other factors besides malignancy, such as smocking or inflammation, may contribute to survival of rectal cancer patients. To the best of our knowledge, the present study is the first report that investigated the prognostic impact of r-CEA in recurrent rectal cancer based on a large cohort.

In summary, serial monitoring of serum CEA after treatment is essential not only for detecting recurrence but also for predicting prognosis after recurrence. Not to mention, monitoring of serum CEA should be done even for tumors with normal pre-CEA because approximately one-fourth of tumors with initially normal CEA show elevated CEA level in recurrent disease.

## MATERIALS AND METHODS

This study was approved by the Institutional Review Board of Samsung Medical Center. The authors retrieved data of 4096 patients treated with curative surgical resection for rectal adenocarcinoma between January 2000 and December 2012 in Samsung Medical Center. Among them, we identified 745 patients who developed any recurrence and had available CEA data.

The cutoff value for upper-normal CEA was 5 ng/ml. Initial CEA and r-CEA was measured within 2 months before treatment or detection of recurrence, respectively. The date of recurrence was defined as the date when clinicians confirmed recurrence and described recommendation for following management in medical records. LRR was defined as any recurrence within the pelvis; DM was defined as any extrapelvic recurrence.

OS was calculated as the interval between date of first recurrence and death or last follow-up. The distribution of categorical variables between groups was analyzed by a chi-square test. Survival rates were calculated by the Kaplan-Meier method and compared by log-rank test for univariate analysis. Multivariate analysis was described by HR and 95% CI derived from a Cox proportional hazards model. A p-value of 0.05 or less was considered statistically significant. SPSS Statistics version 20 (SPSS Inc., an IBM Company, Chicago, IL) was used for analyses.
